# Japanese Kampo Medicine Juzentaihoto Improves Antiviral Cellular Immunity in Tumour-Bearing Hosts

**DOI:** 10.1155/2022/6122955

**Published:** 2022-08-13

**Authors:** Shun Takaku, Masumi Shimizu, Rimpei Morita

**Affiliations:** ^1^Department of Microbiology and Immunology, Nippon Medical School, Tokyo 113-8602, Japan; ^2^Center for Medical Education, Nippon Medical School, Tokyo 113-8602, Japan

## Abstract

Global and antigen-independent immunosuppression by growing tumours can cause life-threating damage when concurrent with an infection in tumour-bearing hosts. In the present study, we investigated whether the oral administration of the Japanese traditional herbal (Kampo) medicine, juzentaihoto (JTT), plays a role in the improvement of antiviral cellular immunity in tumour-bearing hosts. Female BALB/c mice subcutaneously injected with murine colorectal cancer CT26 cells fed a control or JTT diet were inoculated with recombinant vaccinia virus expressing human immunodeficiency virus-1 glycoprotein 160 (vSC25). At 7 days postinfection, anti-vSC25 cellular immunity was evaluated by measuring the abundance of splenic virus-specific CD8^+^ T cells. JTT had no impact on CT26 tumour growth *in vivo*. Surprisingly, JTT augmented anti-vSC25 cellular immunity in CT26-bearing mice. Depletion of either CD25^+^ regulatory *T* (Treg) cells or myeloid-derived suppressor cells (MDSCs) also enhanced anti-vSC25 cellular immunity in tumour-bearing mice but had no therapeutic benefit against tumour growth. However, JTT had no impact on the abundance of these immunosuppressive cells. Overall, our data indicates that JTT contributes to the improvement of anti-vSC25 cellular immunity in tumour-bearing hosts possibly via a mechanism independent of CD25^+^ Treg cells and MDSCs, suggesting that this Kampo medicine can act as a promising antiviral adjuvant in an immunosuppressive state caused by tumours.

## 1. Introduction

Recent advances in cancer therapies have improved the health and survival of cancer patients. In general, cancer patients are in an immunosuppressive state due to the presence of tumours and the expansion of immunosuppressive cells, especially regulatory *T* (Treg) cells and myeloid-derived suppressor cells (MDSCs) [1–3]. Furthermore, such immunosuppression is known to occur globally in tumour-bearing hosts regardless of tumour-antigen specificity [4, 5]. As a result, some viral infections such as flu and SARS-CoV-2 could be fatal for cancer patients. Vaccination is a reasonable strategy to enhance virus-specific immunity in cancer patients. However, genetic mutations, especially in RNA viruses, are known to diminish the antiviral efficacy of vaccination in immunocompromised hosts as well as healthy individuals. In addition, the proliferation of virus-specific T cells is significantly impaired in tumour-bearing hosts [4, 5]. Therefore, a novel strategy to overcome nonspecific immunosuppression in tumour-bearing hosts warrants consideration.

Juzentaihoto (JTT; *Shi-Quan-Da-Bu-Tang* in Chinese and *Sipjeondaebo-Tang* in Korean) is a traditional Japanese herbal (Kampo) medicine. In Japan, JTT is traditionally used as a supportive therapy against anaemia, fatigue, anorexia, scaly skin, and dry mouth [6] as well as more recently to mitigate the side effects of cancer surgery, chemotherapy, and radiotherapy [7–9]. Several studies of murine tumour models have shown that JTT can prevent cancer occurrence [10, 11], suppress metastasis to the liver [12] and lungs [13, 14], and enhance tumour-vaccine efficacy [15]. A recent study by our group revealed that JTT enhanced cluster of differentiation (CD)8-mediated antitumour immunity in CD1d^−/−^ mice with compromised immunosuppression due to the lack of natural killer T cells [16]. JTT is also reported to augment the effects of vaccine against influenza A virus (A/Victoria/210/2009) [17] and *Lactobacillus*-based oral vaccine against human papillomavirus [18]. However, the ability of JTT to enhance antiviral immunity in tumour-bearing hosts has not yet been investigated.

Therefore, the aim of the present study was to investigate the effects of oral administration of JTT in anti-vaccinia cellular immunity in tumour-bearing hosts to evaluate the induction of human immunodeficiency virus (HIV)-1 envelope glycoprotein (gp) 160-derived epitope peptide P18-I10-specific CD8^+^ T cells in mice inoculated with murine recombinant vaccinia virus expressing HIV-1 gp160 (vSC25).

The results of this study showed that in mice subcutaneously injected with CT26 colon carcinoma cells, JTT had no impact on tumour growth in a therapeutic setting. However, the abundance of P18-I10^+^ cells post vSC25 infection was significantly increased in tumour-bearing mice fed a JTT diet as compared to those fed the control diet, whereas such phenomena were not observed in tumour-free mice. Depletion of CD25^+^ Tregs and MDSCs enhanced anti-vSC25 cellular immunity in tumour-bearing mice, although there was no therapeutic gain against the tumour growth. However, JTT had no impact on the abundance of Tregs and MDSCs in tumour-bearing hosts. Overall, these data indicate that JTT can improve anti-vaccinia cellular immunity in tumour-bearing hosts, probably independent of CD25^+^ Tregs and MDSCs, suggesting that JTT is a promising antiviral adjuvant to improve the quality of life of cancer patients.

## 2. Materials and Methods

### 2.1. Animals

Inbred BALB/c mice were purchased from CLEA Japan (Tokyo, Japan) and housed in groups of five in filtered cages in a temperature-controlled, specific pathogen-free animal facility under 12 h light:dark cycle with *ad libitum* access to food and water. Female mice (2-3/group; >6 weeks old) were included in all experiments. The protocols of all animal experiments were approved by the Animal Care and Use Committee of Nippon Medical School (Tokyo, Japan) (Permit No. 30-010) and conducted in accordance with the guidelines for the Care and Use of Laboratory Animals of Nippon Medical School and the “Standards relating to the Care and Keeping and Reducing Pain of Laboratory Animals” as described in the Notice of the Ministry of the Environment (No. 88 of 2006).

### 2.2. Diets Containing Japanese Kampo Medicine JTT

JTT is a dried and powdered hot water extract of 10 crude medicinal herbs/plants mixed at the following ratio: Angelicae radix (3.0), Poria (3.0), Rehmanniae radix (3.0), Ginseng radix (3.0), Cinnamomi cortex (3.0), Paeoniae radix (3.0), Astragali radix (3.0), Glycyrrhizae radix (1.5), Cnidii rhizome (3.0), and Atractylodis lanceae rhizome (3.0) (https://mpdb.nibiohn.go.jp/stork/). JTT was provided as preservative-free pure powder from Tsumura & Co. (Tokyo, Japan). Mice in the experimental group were fed a 2.0% JTT diet, while those in the control group were fed regular chow (type MF diet; 5% fat, 24% protein, and 54% carbohydrate; Oriental Yeast Co., Tokyo, Japan).

### 2.3. Tumour Cell Lines

*N*-nitro-*N*-methylurethane-induced BALB/c murine colon carcinoma CT26 cells were purchased from the American Type Culture Collection (Manassas, VA, USA) and maintained in Roswell Park Memorial Institute1640 complete medium (Thermo Fisher Scientific, Waltham, MA, USA) supplemented with 10% fetal calf serum (FCS) (Merck KGaA, Darmstadt, Germany), penicillin (100 U/ml), streptomycin (100 *μ*g/ml), L-glutamine (2 mM), sodium pyruvate (1 mM), nonessential amino acids, N-2-hydroxyethylpiperazine-N-2-ethane sulfonic acid (10 mM), MEM Vitamin Solution (Thermo Fisher Scientific), and 2-mercaptoethanol (5 × 10^−5^ M).

### 2.4. In Vivo Tumour Assay and Recombinant Vaccinia Virus Inoculation

On day 0, the mice were subcutaneously injected with CT26 cells suspended in Dulbecco's phosphate-buffered saline (DPBS) (Thermo Fisher Scientific) (1 × 10^6^ cells in 200 *μ*l) and fed the control or JTT diet for 21 days postinoculation. The tumour area (length × width [mm]) was measured weekly using a caliper gauge. At 14 days postinjection (dpi), the mice were intraperitoneally (i.p.) inoculated with 1 × 10^7^ plaque-forming units (PFU) of recombinant vaccinia virus expressing HIV-1 envelope gp160 of IIIB isolate (vSC25) [19]. In a subset of experiments, some mice were also i.p. injected with 0.3 mg of anti-mouse CD25 monoclonal antibody (mAb) (clone PC-61.5.3, catalogue number: BE0012; Bio X Cell, West Lebanon, NH, USA) on 14 dpi or with 0.2 mg of anti-mouse Ly6G/Ly6C (Gr-1) mAb (clone RB6-8C5, catalogue number: BE0075; Bio X Cell) on 13 and 14 dpi with tumours. At 7 dpi with vSC25, the mice were sacrificed, and the spleens were collected to evaluate anti-vSC25 cellular immunity and the abundance of the immunosuppressive cells, such as CD4^+^ CD25^+^ Foxp3^+^ Tregs and MDSCs.

### 2.5. Flow Cytometry

To determine the surface molecule expression patterns of the cells, flow cytometry was performed using a BD FACSCanto™ II flow cytometry system (BD Immunocytometry Systems, San Jose, CA, USA). In brief, spleen cells were washed with 2 ml of cold DPBS, then stained with diluted Zombie NIR^™^ dye (1 : 500 in DPBS; BioLegend, San Diego, CA, USA) at room temperature in the dark for 15 min and washed again with DPBS with 2% heat-inactivated FCS and 0.1% sodium azide (FACS buffer). To reduce nonspecific Ab binding to Fc receptors, the cells were incubated with 0.5 *μ*g of anti-CD16/CD32 Ab (clone 2.4G2, catalogue number: 14-0161; eBioscience, Inc., San Diego, CA, USA) at 4°C for 15 min and then stained with secondary Abs for 30 min at 4°C. Subsequently, the cells were washed once with 2 ml of FACS buffer prior to analysis. The following Abs were used in this study (all purchased from BioLegend): phycoerythrin (PE)/cyanine dye (Cy)7-conjugated anti-CD3 (clone 17A2, catalogue number: 100220), fluorescein isothiocyanate (FITC)-conjugated anti-CD4 (clone GK1.5, catalogue number: 100405), FITC-conjugated anti-CD8*β* (clone YTS156.7.7, catalogue number: 126606), allophycocyanin (APC)-conjugated anti-CD25 (clone 3C7, catalogue number: 101909), PE/Cy7-conjugated anti-CD11b (clone M1/70, catalogue number: 101215), APC-conjugated anti-CD45 (clone 30-F11, catalogue number: 103112), APC/Cy7-conjugated anti-CD45 (clone 30-F11, catalogue number: 103116), FITC-conjugated anti-Ly6G (clone 1A8, catalogue number: 127606), and PE-conjugated anti-Ly6C (clone HK1.4, catalogue number: 128007). To evaluate anti-vSC25 cellular immunity, the PE-conjugated HIV-1 P18-I10 peptide-loaded H-2D^d^ tetramer was purchased from Medical and Biological Laboratories Co., Ltd. (Nagoya, Japan).

Tregs were identified by positive staining for Foxp3, CD3, CD4, and CD25. After washing once with 2 ml of cold FACS buffer, the Tregs were fixed with 1 ml of eBioscience ™ Intracellular Fixation & Permeabilization Buffer (eBioscience, Inc.) at 4°C for 30 min, then washed twice with 2 ml of 1 × eBioscience ™ Permeabilization Buffer (eBioscience, Inc.) and stained with 0.4 *μ*g of PE-conjugated anti-Foxp3 (clone FJK-16s, catalogue number: 12-5773-80; eBioscience, Inc.) at 4°C for 30 min, followed by two additional washes with 2 ml of 1 × eBioscience ™ Permeabilization Buffer. For each sample, 3000–10000 events were acquired. The obtained data were analysed using FlowJo software (version 9.3.1; Tree Star, Inc., Ashland, OR, USA).

### 2.6. Statistical Analysis

Data were analysed using Kruskal–Wallis test with Dunnett's multiple comparisons post hoc test, Student's *t* test, or nonparametric Mann–Whitney *U* test with GraphPad Prism 6 software (version 6.0 d; GraphPad Software, La Jolla, CA, USA). A probability (*p*) value of <0.05 was considered statistically significant.

## 3. Results

### 3.1. Oral Administration of JTT Enhances Anti-Vaccinia Cellular Immunity in Tumour-Bearing Mice

**Figure 1:**
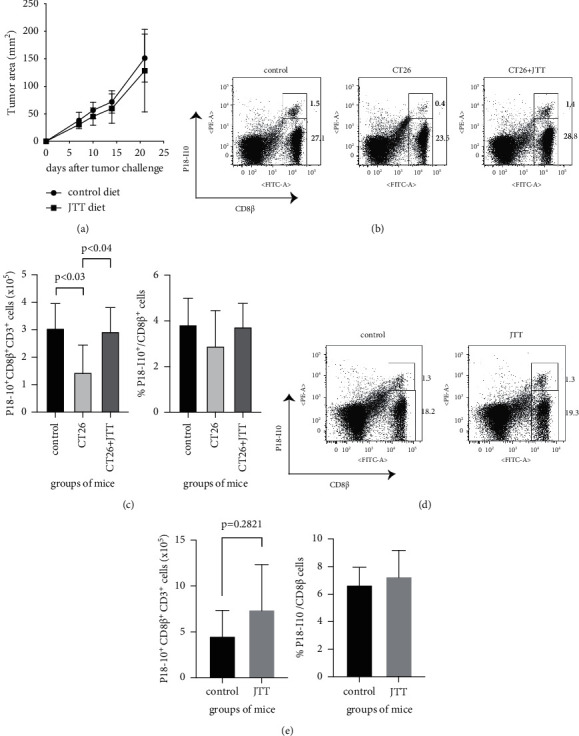
JTT enhances anti-vaccinia cellular immunity in tumour-bearing mice but not in naïve mice after vSC25 inoculation. BALB/c mice were subcutaneously challenged with 1 × 10^6^ syngeneic CT26 cells and subsequently fed with either the control or JTT diet for 21 days. The tumour area was measured once or twice per week using a caliper gauge until 21 days post-tumour exposure. The measurements were calculated as tumour length (mm) × width (mm). Each experiment was conducted using 2-3 mice. The results of two independent experiments were pooled for analysis (a). On the day of the experiment of (a), we added the control diet group without a tumour challenge. Furthermore, in other experiments, the tumour-free BALB/c mice were fed a control or JTT diet for 21 days. Fourteen days after the tumour challenge (14 days after the assigned diet feeding), mice were i.p. inoculated with 1 × 10^7^ PFU of recombinant vaccinia (vSC25). On day 7 post vaccinia infection, spleen cells were isolated from mice and stained with Abs against CD3, CD8*β*, and HIV-1 P18-I10 peptide-loaded H-2D^d^ tetramer. Flow cytometry was performed to determine the proportions of CD3^+^ CD8*β*^+^ H-2D^d^/P18-I10^+^ cells to evaluate anti-vaccinia cellular immunity. (b) and (d) show representative flow data. The presented dot plots were obtained by gating the CD3^+^ population. (c) and (e) show the cumulative data of two independent experiments (*n* = 5 for the control group, *n* = 6 for the CT26 group, and *n* = 5 for the CT26 + JTT group in (c); *n* = 5 for the control group and n = 6 for the JTT group in (e)). Data are expressed as mean ± standard deviation (SD). *p* < 0.03 between the control and CT26 groups, and *p* < 0.04 between the CT26 and CT26 + JTT groups by Kruskal–Wallis test with Dunnett's multiple comparison test.

The results of our previous study revealed that JTT had no effect on the proliferation of CT26 cells in wild-type BALB/c mice in a prophylactic setting [16, 20]. Thus, we hypothesised that JTT would have no impact on tumour growth in a therapeutic setting. To test this hypothesis, naïve BALB/c mice were fed a control or JTT diet for 3 weeks post-subcutaneous injection of 1 × 10^6^ CT26 cells. As expected, there was no difference in tumour area between the control and JTT diet groups (Figure 1(a)). Based on these findings, the effect of JTT on anti-vaccinia cellular immunity was evaluated in tumour-bearing hosts. In brief, after 14 days of feeding with the control or JTT diet, tumour-bearing mice were i.p. inoculated with 1 × 10^7^ PFU of vSC25. We also added naïve (tumour-free) mice treated with vSC25 as a control to demonstrate that tumour burden suppressed anti-vaccinia cellular immunity. At 7 dpi, the abundance of splenic CD8^+^ HIV-1 P18-I10 peptide-loaded H-2D^d^ tetramer-positive cells was measured to evaluate anti-vaccinia cellular immunity since this epitope peptide is derived from vSC25. As shown in Figures 1(b) and 1(c) (left), the total number of P18-I10^+^ cells was significantly reduced in the tumour-bearing mice compared with the naïve mice, whereas this reduction was markedly recovered in the tumour-bearing mice fed with JTT diet. However, there was no difference among these three mice groups in the frequency of P18-I10^+^ cells in CD8^+^ T cells (Figures 1(b) and 1(c) right). We also evaluated the abundance of P18-I10^+^ CD8^+^ T cells in vSC25-treated naïve mice fed with control or JTT diet. Interestingly, JTT had no impact on the abundance of tetramer-positive cells in naïve mice post-vSC25 infection (Figures 1(d) and 1(e)). These data suggest that JTT improved anti-vaccinia cellular immunity that was suppressed by tumour burden.

### 3.2. Depletion of Tregs, despite the Lack of Antitumour Effects, May Enhance Anti-Vaccinia Cellular Immunity in Tumour-Bearing Mice

**Figure 2:**
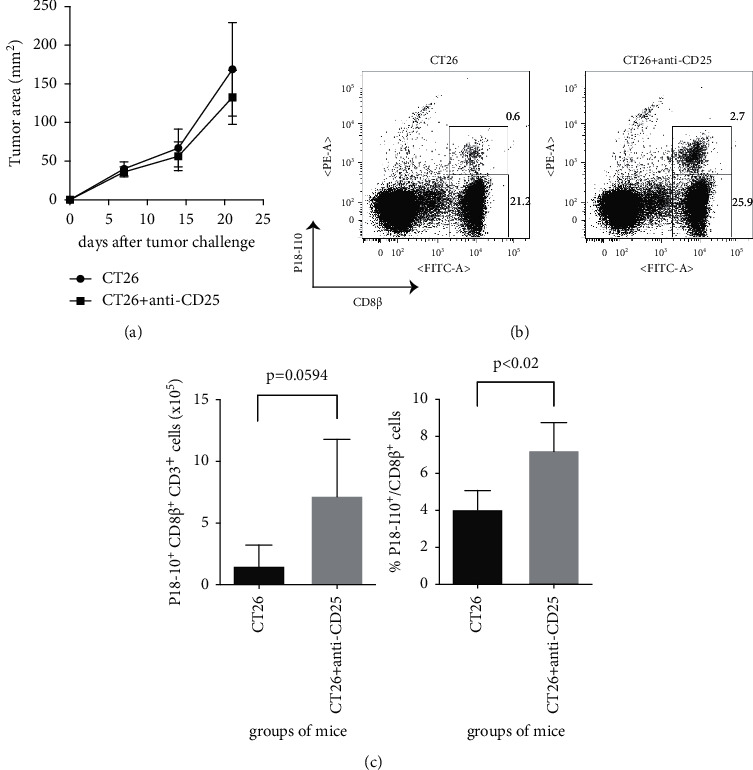
Depletion of Tregs, despite the lack of antitumour effects, may enhance anti-vaccinia cellular immunity in tumour-bearing mice. BALB/c mice were subcutaneously challenged with 1 × 10^6^ syngeneic CT26 cells. On day 14 after tumour exposure, mice were i.p. inoculated with 1 × 10^7^ PFU of recombinant vaccinia (vSC25). Simultaneously, some mice were also i.p. administered with 0.3 mg of anti-CD25 mAb. At 7 dpi with vSC25, the recovered splenocytes were subsequently stained with Abs against CD3, CD8*β*, and HIV-1 P18-I10 peptide-loaded H-2D^d^ tetramer. Flow cytometry was performed to determine the proportions of CD3^+^ CD8*β*^+^ H-2D^d^/P18-I10^+^ cells. Tumour area is shown in (a). (b) shows representative flow data. The presented dot plots were obtained by gating the CD3^+^ population. (c) shows the cumulative data of two independent experiments (*n* = 4 for the CT26 group and *n* = 4 for the CT26 + anti-CD25 group). Data are expressed as mean ± standard deviation (SD). *p*=0.0594 (Student's *t* test) in the left panel of (c). *p* < 0.02 (Student's *t* test) in the right panel of (c).

CD4^+^ CD25^+^ Foxp3^+^ Tregs are a key regulator of antiviral immunity [21–26]. Thus, the ability of Tregs to suppress anti-vaccinia immunity of tumour-bearing mice was investigated. In brief, at 14 dpi, Tregs were depleted from tumour-bearing mice by a single inoculation of an anti-CD25 Ab immediately prior to an injection of vSC25. Our group and others have previously reported that the prophylactic abrogation of Tregs with an anti-CD25 Ab strongly suppressed the growth of CT26 cells *in vivo* [27, 28]. However, depletion of Tregs at 14 dpi had no effect on tumour growth, in agreement with a previous report [29] (Figure 2(a)). Nonetheless, there was not only a trend toward an increase in the total number of P18-I10^+^ CD8^+^ T cells in tumour-bearing mice treated with the anti-CD25 Ab, although not statistically significant (*p*=0.0594), but there was also a significant increase in the frequency of P18-I10^+^ cells among CD8^+^ T cells in these mice (Figures 2(b) and 2(c)). These data indicate that the depletion of Tregs, despite the lack of any therapeutic antitumour effect, may still enhance anti-vaccinia cellular immunity in tumour-bearing mice, suggesting that Tregs may suppress anti-vaccinia immunity in the presence of established tumours *in vivo*.

### 3.3. Depletion of MDSCs, despite the Lack of Antitumour Effects, Enhances Anti-Vaccinia Cellular Immunity in Tumour-Bearing Mice

**Figure 3:**
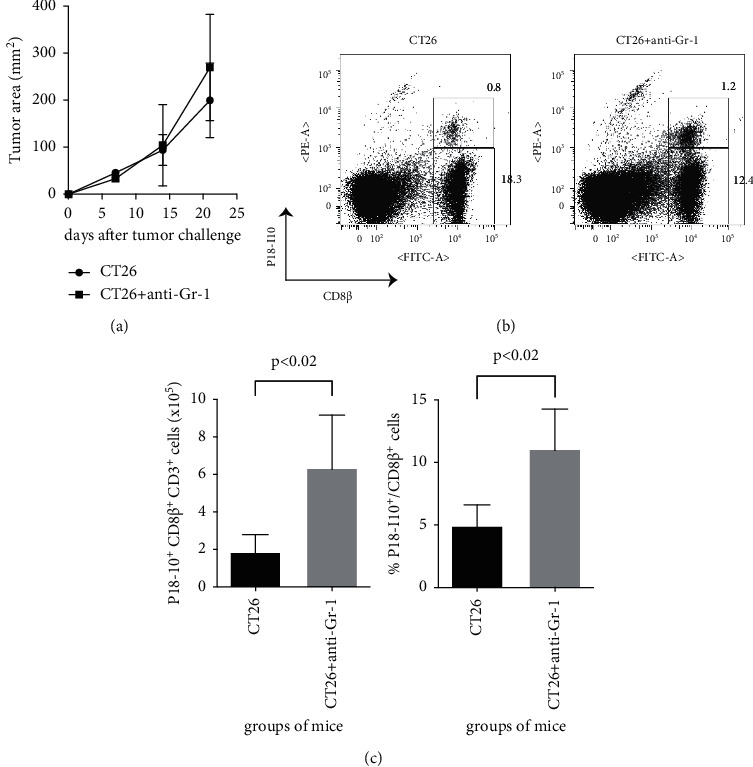
Depletion of MDSCs, despite the lack of antitumour effects, enhances anti-vaccinia cellular immunity in tumour-bearing mice. BALB/c mice were subcutaneously challenged with 1 × 10^6^ syngeneic CT26 cells. Some mice were also i.p. administered with 0.2 mg of anti-Gr-1 mAb on days 13 and 14 after tumour exposure. All mice were i.p. inoculated with 1 × 10^7^ PFU of recombinant vaccinia (vSC25) on day 14. At 7 dpi with vSC25, the recovered splenocytes were subsequently stained with Abs against CD3, CD8*β*, and HIV-1 P18-I10 peptide-loaded H-2D^d^ tetramer. Flow cytometry was performed to determine the proportions of CD3^+^ CD8*β*^+^ H-2D^d^/P18-I10^+^ cells. Tumour area is shown in (a). (b) shows representative flow data. The presented dot plots were obtained by gating the CD3^+^ population. (c) shows the cumulative data of two independent experiments (*n* = 5 for the CT26 group and *n* = 4 for the CT26 + anti-Gr-1 group). Data are expressed as mean ± SD. *p* < 0.02 (Mann–Whitney test) in the left and right panel of (c).

MDSCs are also a key regulator of antiviral immunity in immune-competent hosts [30, 31]. Thus, the effect of MDSCs in suppression of anti-vaccinia immunity in tumour-bearing mice was investigated. In brief, MDSCs were depleted in tumour-bearing mice with an anti-Gr-1 Ab, which was followed by a challenge with vSC25. Although the anti-Gr-1 Ab had no antitumour therapeutic effect *in vivo* (Figure 3(a)), the total number of P18-I10^+^ CD8^+^ T cells and the frequency of P18-I10^+^ cells among CD8^+^ T cells in the spleen were significantly increased in Gr-1-treated mice post-vSC25 infection (Figures 3(b) and 3(c)). These data indicate that abrogation of MDSCs, despite the lack of any therapeutic antitumour effect, enhanced anti-vaccinia cellular immunity in tumour-bearing mice, suggesting that MDSCs suppress anti-vaccinia immunity in the presence of established tumours *in vivo*.

### 3.4. JTT Had No Impact on the Numbers of Tregs and MDSCs in Tumour-Bearing Mice

**Figure 4:**
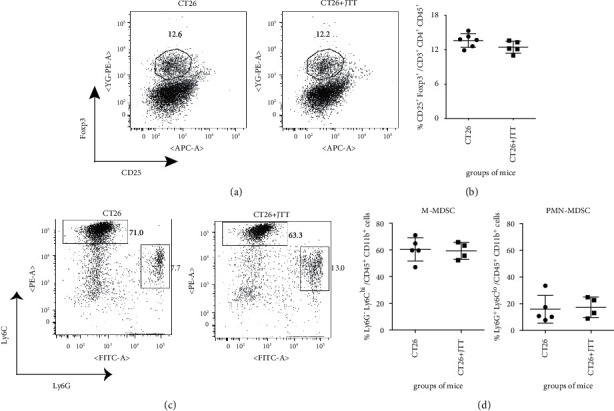
JTT has no impact on the abundance of CD4^+^ CD25^+^ Foxp3^+^ Tregs and MDSCs in tumour-bearing mice after vSC25 inoculation. BALB/c mice were subcutaneously challenged with 1 × 10^6^ syngeneic CT26 cells and subsequently fed with either the control or JTT diet for 21 days. On day 14 after tumour injection, mice were i.p. inoculated with 1 × 10^7^ PFU of vSC25. At 7 dpi, the spleens were collected from the mice and recovered cells were subsequently stained with Abs against CD3, CD4, CD25, and Foxp3 (a and b) or against CD45, CD11b, Ly6G, and Ly6C (c and d). Flow cytometry was performed to measure the proportions of CD25^+^ Foxp3^+^ cells. The presented dot plots in (a) were obtained by gating the CD3^+^ CD4^+^ population. Flow cytometry was also performed to measure the abundance of Ly6G^−^ Ly6C^hi^ M-MDSCs and Ly6G^+^Ly6C^lo^ PMN-MDSCs. The presented dot plots in (c) were obtained by gating the CD45^+^ CD11b^+^population. (b) and (d) show cumulative data of two independent experiments. Each group consisted of 2-3 mice. Each symbol corresponds to one data point. The data are expressed as mean ± SD.

As Treg depletion may enhance anti-vaccinia cellular immunity in tumour-bearing mice (Figures 2(b) and 2(c)), the potential role of JTT in reducing the number of Tregs was investigated by comparing the number of CD4^+^ CD25^+^ Foxp3^+^ Tregs in the spleens derived from tumour-bearing mice fed with a control or JTT diet for a total of 21 days (7 dpi with vSC25). We found no difference in the abundance of Tregs in tumour-bearing mice between the two diet groups (Figures 4(a) and 4(b)).

As the abrogation of MDSCs enhanced anti-vaccinia cellular immunity in the presence of established tumours (Figures 3(b) and 3(c)), the potential role of JTT in reducing the number of MDSCs in tumour-bearing mice was investigated. MDSCs can be divided into two types: CD11b^+^ Ly6G^−^ Ly6C^hi^ monocytic (M)-MDSCs and CD11b^+^ Ly6G^+^ Ly6C^lo^ polymorphonuclear (PMN)-MDSCs [32]. Thus, the numbers of splenic M-MDSCs and PMN-MDSCs derived from vSC25-inoculated tumour-bearing mice in the control and JTT diet groups were compared. As shown in Figures 4(c) and 4(d), there were no differences in the numbers of M-MDSCs and PMN-MDSCs between the groups. Taken together, these findings suggest that JTT had no impact on the numbers of Tregs and MDSCs in tumour-bearing mice post-vSC25 inoculation, suggesting that the augmentation of anti-vaccinia cellular immunity by JTT in tumour-bearing hosts may be independent of Tregs and MDSCs.

## 4. Discussion

The results of the present study demonstrated that tumour burden suppressed anti-vaccinia cellular immunity and that the oral administration of JTT improved the impairment of antiviral CD8^+^ T cell immunity in tumour-bearing mice (Figures 1(b) and 1(c) (left)), but not in immune-competent tumour-free mice (Figures 1(d) and 1(e)). Previous studies have reported that some Japanese Kampo medicines including JTT can enhance antiviral humoral and/or cellular immunity in various settings [17, 18, 33]. However, the impact of Kampo medicine on antiviral immunity in tumour-bearing hosts in a potentially immunosuppressive state remains unclear. To the best of our knowledge, this is the first report to show that JTT improved antiviral cellular immunity in tumour-bearing immunosuppressive hosts. Although controversial, JTT has reportedly demonstrated anticancer effects in various murine tumour models [6, 10, 11, 14, 16]. JTT had no impact on tumour growth *in vivo* in the current study (Figure 1(a)), but it did produce an antiviral adjuvant effect in tumour-bearing mice, suggesting that JTT could be prescribed to cancer patients to alleviate acute viral infections and avoid potentially life-threating infections.

CD4^+^ CD25^+^ Foxp3^+^ Tregs were the predominant immunosuppressive cells and conveyed the antitumour T cell effector functions in the subcutaneous CT26 tumour BALB/c mouse model used in this study. Actually, prophylactic depletion of Tregs is reported to contribute to near complete eradication of CT26 cells *in vivo* [27, 28, 34, 35]. However, abrogation of Tregs had no impact on established tumours in the present study (Figure 2(a)). Nonetheless, depletion of Tregs may still have played a role in the enhanced anti-vaccinia cellular immunity of the tumour-bearing mice (Figures 2(b) and 2(c)), suggesting that tumour-specific, as compared to nonspecific, cellular immunity may be strongly suppressed in cancer hosts.

The role of Tregs in viral immunology is context-dependent. In the context of chronic infections with HIV and hepatitis B and C viruses, Tregs are reported to suppress protective immune responses and subsequently diminish virus elimination [25, 26]. On the contrary, Tregs play beneficial roles in acute viral infections with herpes simplex virus type 1, West Nile virus, and influenza virus, which dysregulate the immune response against viral infection, resulting in greater severity of the disease [24]. Although Tregs appear to play a role in suppressing excessive immune responses against acute viral infection, abrogation of these cells may be beneficial to maintaining antiviral immunity in the immunosuppressive state of tumour-bearing hosts.

A recent study revealed that MDSCs are a major obstacle to antitumour immunity and numerous immunotherapies [36]. Actually, the depletion of MDSCs with an anti-Gr-1 Ab is reported to prevent tumour recurrence in a mouse model [37]. MDSCs also regulate natural killer and T cell responses after vaccinia virus infection in immunocompetent mice [30, 31]. In the present study, abrogation of MDSCs with an anti-Gr-1 Ab failed to reduce the tumour burden (Figure 3(a)), but yet enhanced anti-vaccinia cellular immunity in tumour-bearing mice (Figures 3(b) and 3(c)). MDSCs, especially M-MDSCs, contribute to the suppression of excessive inflammation caused by acute infection to inhibit virus-specific responses of CD8^+^ T cells [31]. However, in the context of an immunosuppressive state, as with cancer patients, antiviral immune responses could be insufficient to resolve acute viral infections due to the existence of MDSCs. Therefore, it would be beneficial to regulate M-MDSCs to restore antiviral immunity in immunosuppressive, but not immunocompetent, hosts. These findings warrant further investigation.

Although both CD25^+^ Tregs and MDSCs suppressed anti-vaccinia cellular immunity in tumour-bearing mice, JTT had no impact on the abundance of these immune-regulatory cells (Figure 4). These data demonstrate that augmentation of antiviral immunity by JTT may be independent of Tregs and MDSCs. Since JTT is considered as a relatively safe medicine in East Asia, including Japan, the manipulation of Tregs or MDSCs with JTT presents a promising strategy to enhance antiviral immunity in tumour-bearing hosts.

A standard research strategy in the field of infectious diseases is to focus on the actual pathogen via the development of vaccines and pathogen-specific medicines, such as neuraminidase inhibitors against influenza viruses and protease-inhibitors against HIV. However, various genes of RNA viruses are known to easily mutate mainly due to the lack of repair enzymes, which often leads to failure of virus-specific therapies. Therefore, it would be beneficial to focus on the status of the infected hosts rather than the pathogen. Actually, traditional herbal medicines including Kampo medicine have been used for more than 1000 years in East Asia to restore physical strength and energy. Thus, the findings of this study suggest that future studies should consider focusing on the status of the host, rather than a particular pathogen.

## 5. Conclusions

In conclusion, to the best of our knowledge, the present study is the first to demonstrate that JTT enhanced anti-viral cellular immunity in tumour-bearing hosts, thereby providing novel insights into the use of JTT as antiviral adjuvant in tumour-bearing hosts in an immunosuppressive state.

## Data Availability

All data generated or analysed during this study are included in this published article.

## Abbreviations

JTT: Juzentaihoto
vSC25: Recombinant vaccinia virus expressing HIV-1 gp160
Treg: Regulatory T cells
MDSCs: Myeloid-derived suppressor cells
M-MDSCs: Monocytic MDSCs
PMN-MDSCs: Polymorphonuclear MDSCs.
